# Epithelioma, Extirpated by Blood Injections

**Published:** 1898-07

**Authors:** 


### Epithelioma, Extirpated by Blood Injections.

Henry S——, Stamford; American; age 41; first seen February 4, 1898. Epithelioma of the right ear, involving the lobe, of three months standing. Had been treated unsuccessfully with the new specific called eryselapine, under which it had gradually grown worse. On the 6th, five minims absolute alcohol with twenty minims pure bovinine were injected into the growth and into the line of demarcation all around between the healthy and diseased tissues. Externally, it was dressed with iodoform-bovinine three times a day. The injection was repeated February 9th, 14th, 24th, 28th, and March 3d, with the iodoform-bovinine dressing changed three times every day. March 8th, the epithelioma had separated itself from the healthy tissue almost entirely; and on the 9th I removed it by simply plucking it out with a thumb forceps, leaving a healthy granulating surface in its seat. About two-thirds of the lobe was involved in the epitheliomatous growth and came away in it. This is a case of exceedingly great interest, and will be closely watched, and if any further growth appears, the treatment and result will be reported.—*Sound View Hospital Reports.*

